# Transparent peer review for all

**DOI:** 10.1038/s41467-022-33056-8

**Published:** 2022-10-19

## Abstract

Starting in 2016, we have offered authors the option to publish the comments received from the reviewers and their responses alongside the paper. As we believe that transparency strengthens the quality of peer review, we are now moving to publish the exchanges between authors and reviewers for all research articles submitted from November 2022 onward and accepted for publication. Referees will still have the option to remain completely anonymous, to sign their reports, and/or to choose to be acknowledged by name as part of our reviewer recognition scheme.

The evaluation of scientific studies by peers prior to their publication is an important tool to ensure that widely disseminated science adheres to sufficiently rigorous standards of proof, and that the reported findings are adequately interpreted. Amongst other benefits, the peer review process can help to improve the reliability of early results and the overall value of published manuscripts. For a long time, this critical component of the scientific endeavor was hidden from view – only witnessed by the authors, editors, and referees involved in evaluating a particular submission. Over the past two decades however, this shroud of secrecy has progressively been lifted, as various ‘Open Review’ processes are being implemented at a growing number of journals^1,2^.

In 2016, *Nature Communications* provided authors with the option to publish peer reviewers’ comments and their responses alongside their paper^3^. Since then, similar options are being offered by several journals from the Nature portfolio. *Nature* itself has been offering this option as a pilot since February 2020, and nearly half of all papers published in 2021 were accompanied by peer review reports^4^. At *Nature Communications*, the opt in rate for the same period was approximately 70%.

A few years ago, in a comment published in *Nature* entitled ‘Publish peer reviews’ the authors made a strong case for making peer review files part of the official scientific record^1^. Amongst the listed benefits of an open review process, the idea that the peer review comments represent valuable scholarship that should be preserved is one we particularly agree with. As editors, we also feel that in some cases – where authors and referees could not reach complete agreement on how certain data should be interpreted – making reports and the response from the authors public shows that valid potential caveats or limitations of the study have been raised and discussed. Peer review, in itself, cannot completely guarantee the validity of the conclusions reached by authors, especially in situations where competing hypotheses exist. Armed with the peer review file, each reader can better critically assess the robustness of the conclusions. Making the back and forth between authors and referees available also benefits authors, as it allows them to bring forward arguments in support of their view that might otherwise be difficult to logically integrate within a paper’s narrative.

“*Amongst the listed benefits of an open review process, the idea that the peer review comments represent valuable scholarship that should be preserved is one we particularly agree with*”

In short, we strongly believe that publishing peer review files greatly benefits scientists as well as the public at large, serves to increase confidence in the peer review process, and augments the value of peer reviewed work. It also provides greater recognition to reviewers should they wish to be acknowledged for their contribution. The data indicates that a significant majority of our authors already agree, and it is our hope that those who had not considered it previously will realize its benefits. Hence, for manuscripts received from November 2022 onwards, the peer review files will be published alongside every published *Nature Communications* research article.

We are very grateful for the time our reviewers dedicate to provide constructive comments to our authors, and we appreciate that they may wish to remain anonymous; therefore, for our referees, signing their reports – or choosing to be acknowledged by name as part of our reviewer recognition scheme – will remain optional. We will also continue to offer our authors the possibility to opt-in double blind peer-review, during which their identities remain anonymous to the referees during the review process.

Finally, we hope that Early Career Researchers from all fields of science all over the world – now being able to read the exchange between authors and reviewers for all our papers – will benefit from getting first hand insight into the inner workings of the peer review process.
